# Adaptability and High School Students’ Online Learning During COVID-19: A Job Demands-Resources Perspective

**DOI:** 10.3389/fpsyg.2021.702163

**Published:** 2021-08-17

**Authors:** Andrew J. Martin, Rebecca J. Collie, Robin P. Nagy

**Affiliations:** School of Education, University of New South Wales, Sydney, NSW, Australia

**Keywords:** adaptability, job demands-resources, online learning, remote instruction, COVID-19, achievement

## Abstract

The present study investigated the role of adaptability in helping high school students navigate their online learning during a period of COVID-19 that entailed fully or partially remote online learning. Drawing on Job Demands-Resources theory and data from a sample of 1,548 Australian high school students in nine schools, we examined the role of adaptability in predicting students’ online learning self-efficacy in mathematics and their end of year mathematics achievement. It was found that beyond the effects of online learning demands, online and parental learning support, and background attributes, adaptability was significantly associated with higher levels of online learning self-efficacy and with gains in later achievement; online learning self-efficacy was also significantly associated with gains in achievement—and significantly mediated the relationship between adaptability and achievement. These findings confirm the role of adaptability as an important personal resource that can help students in their online learning, including through periods of remote instruction, such as during COVID-19.

## Introduction

The COVID-19 pandemic led to an unexpected and rapid shift to remote learning for students around the world. In the space of a few weeks, the very nature of learning and instruction was transformed (Australian Academy of Science, 2020). Learning and instruction moved to remote online modes at speed and scale. The extent to which students have successfully responded and adjusted to these disruptions has been key to how they have coped academically (Australian Academy of Science, 2020). This being the case, *adaptability* may be a personal attribute that is highly relevant through times of online remote learning and instruction, such as during COVID-19 and any other future periods of disrupted learning.

Adaptability is the capacity to regulate one’s behaviors, thoughts, and feelings in response to novel, variable, uncertain, and unexpected situations and circumstances (Martin et al., 2012, 2013). Adaptability has been identified as an important capacity for students’ academic and personal development, including their motivation, engagement, achievement, and social-emotional wellbeing (Martin et al., 2013;Holliman et al., 2018, 2019, 2021). Given adaptability is specifically aimed at successfully navigating change, uncertainty, and novelty, it is also likely a vital personal attribute to support students during periods of novelty, variability, and uncertainty, such as with COVID-19 restrictions and lockdowns, including periods of online learning through these times. To the extent that adaptability is associated with positive educational processes and outcomes during online learning, it may be an important area of focus for educational interventions.

**Figure 1:**
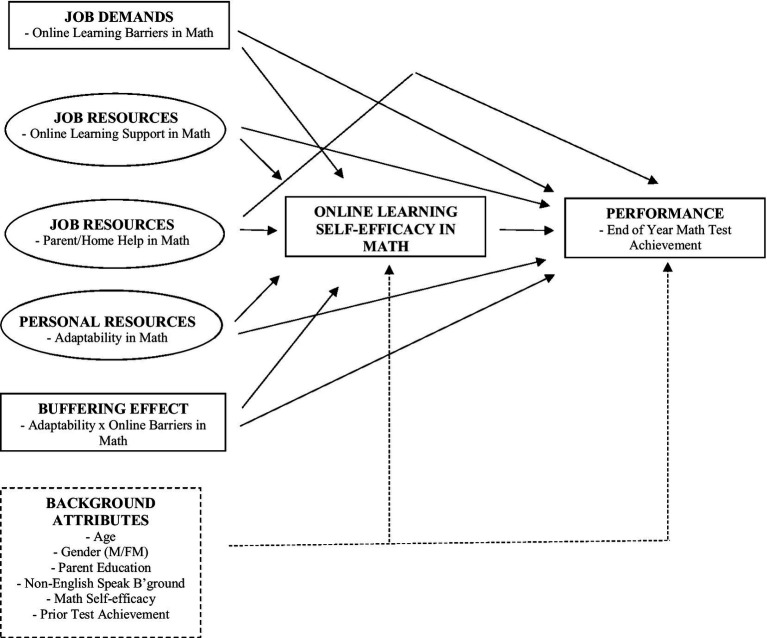
Hypothesized JD-R process in online mathematics.

The aim of this research was to expand current knowledge of adaptability by focusing on its role in students’ academic development and online learning during a period of COVID-19 that entailed fully or partially remote online learning. Drawing on Job Demands-Resources theory (JD-R theory; Bakker and Demerouti, 2017, 2018) and focusing on learning and instruction in mathematics, we examined the role of adaptability in predicting students’ online learning self-efficacy and their end of year achievement. We were particularly interested in the extent to which adaptability (a personal resource) played a role in students’ online learning self-efficacy and achievement beyond the effects of any online learning demands, online and parental learning support, and background attributes. Figure 1 demonstrates the hypothesized model under examination.

## Theoretical Background and Literature Review

### Adaptability

As described above, adaptability is the capacity to adjust behaviors, thoughts, and feelings in response to novel, variable, uncertain, and unexpected situations and circumstances (Martin et al., 2012, 2013). It is thus a tripartite perspective composed of behavioral, cognitive, and emotional dimensions (Martin et al., 2012, 2013). Research among school students has demonstrated links between adaptability and students’ engagement and achievement (Martin et al., 2012, 2013; Collie et al., 2017), identified the role of adaptability in young people’s responses to climate change (Liem and Martin, 2015), demonstrated the role of adaptability in reducing students’ failure dynamics (Martin et al., 2015), shown links with university students’ engagement and longer-term achievement (Holliman et al., 2018), and validated adaptability across diverse international contexts (Martin et al., 2017). There is, then, a strong evidence base for the role of adaptability in students’ positive development. The present study is an opportunity to expand on this by investigating the role of adaptability in assisting students’ online learning experiences and outcomes during a period of substantial novelty, variability, and uncertainty—specifically, online learning during the COVID-19 pandemic. Given there is likely to be substantial novelty, variability, and uncertainty ahead due to the evolving nature of the pandemic (Australian Academy of Science, 2020), it is important to identify modifiable psycho-behavioral attributes that may assist students through this and through future periods of disrupted learning. The present study focuses on adaptability as one such attribute.

### Online Learning and Instruction

Online learning encompasses the use of desktop computers, laptops, tablets, virtual reality devices, mobile phones, personal digital assistants, and more (Sung et al., 2017). Online learning methods traverse staged programs of instruction, animation, gaming, simulations, video instruction, collaborative documents, chatrooms, etc. There are also many content and learning management systems (e.g., Canvas, Moodle, and Blackboard) that facilitate online learning. Online learning activity predominantly comprises synchronous instruction that is in real-time (such as live video interaction) and asynchronous instruction that may be pre-recorded or a standalone self-paced online program (Thalheimer, 2017).

When appraising the effectiveness of online learning, there is a mixed evidence base. On the positive side, there is meta-analytic evidence demonstrating the effectiveness of various online learning approaches, yielding generally small to moderate effect sizes (Yuwono and Sujono, 2018). There is also meta-analytic evidence that mobile-computer-supported learning can enhance collaborative learning (Sung et al., 2017). On the negative side, there is research suggesting that online learning approaches are not as effective as real-time in-class learning. For example, Clinton (2019); see also Delgado et al. (2018) found that students reading material in paper-based form showed greater comprehension than students reading the same material in digital form. Findings from PISA 2012 (Peña-López, 2015) found that students who used computers very frequently at school performed more poorly than students with other levels of computer use. Moreover, it seems that many teachers are not highly trained in harnessing technology to help students learn (Peña-López, 2015). There is also a line of research demonstrating generally null or minimal effects when comparing online and in-class modes. In an online coaching program for teachers, there were no significant effects for student achievement (Kraft and Hill, 2020). In a study of online distance education, Cavanaugh et al. (2004) found comparable student achievement across online and in-class instructional modes.

One reason why there are such mixed findings is because there are many factors that are implicated in the success of online modes. Factors related to technology access, technology skills, instructional and resource quality, parent/home support, ethnicity, socioeconomic status, and learning support needs have all been identified as influencing the extent to which online learning is effective or not (AITSL, 2020; Australian Academy of Science, 2020). Importantly, however, given the substantial novelty, variability, and uncertainty associated with online learning during COVID-19, it is also likely that various personal psychological attributes have potential to assist students’ learning during this time and in future periods of disrupted learning. Adaptability is hypothesized as one such factor and is the focus of our investigation into online learning experiences during a period of COVID-19 in Australia when students were variously engaged in fully or partially remote online learning.

### Job Demands-Resources Theory

We draw on JD-R as a means to explore and understand the role of adaptability in students’ online learning experiences during COVID-19. Before doing so, we summarize JD-R as traditionally formulated in workplace research. Then, we extrapolate from this to explore its relevance to students’ online learning and to frame the present study.

#### Job Demands-Resources Theory in the Workplace

Job Demands-Resources theory holds that there are specific contextual factors in jobs and work roles that help or hinder employees’ outcomes (Schaufeli and Bakker, 2004). Job demands are aspects of work that require psychological or physical exertion (e.g., performing under a heavy workload and addressing mounting deadlines) and that are linked with psychological or physical costs (e.g., poor mental and physical health aspects of burnout; Bakker and Demerouti, 2017; Collie et al., 2020a). Job resources are aspects of work that help employees attain desired work-related goals and growth (e.g., peer support; Demerouti et al., 2001) and are linked with positive outcomes (e.g., motivation and health; Skaalvik and Skaalvik, 2018).

In recent years, JD-R theory has recognized that there are also personal resources that determine employees’ work-related functioning (Xanthopoulou et al., 2007; Collie et al., 2020a). Personal resources are modifiable, personal capacities that reflect an individual’s potential to influence their working environment; similar to job resources, personal resources are linked with positive outcomes (Schaufeli and Taris, 2014). Collie et al. (2020a); see also Granziera et al. (2021) proposed that adaptability can be considered a personal resource, as it is a modifiable capacity that can help an individual navigate change in the workplace and effect positive outcomes.

In addition to these “main effects” of demands and resources, there is also a buffering possibility suggested by JD-R theory (Bakker and Demerouti, 2017)—and adaptability may be an important part of this. For example, Granziera et al. (2021) proposed that adaptability may buffer the negative effects of job demands such that employees high in adaptability are less likely to experience the negative effects of job demands. Granziera et al. (2021) demonstrated support for this by showing that adaptability offset the negative effect of role conflict on emotional exhaustion in teachers (see also Dicke et al., 2018).

Alongside the need to consider potential buffering effects, we also draw attention to more recent refinements of JD-R theory that speak to how demands and resources may be perceived differently by individuals: A given job demand or job resource may be perceived in different ways by different people—not all individuals perceive a demand as a hindrance and not all individuals perceive a resource as a help (Bakker and Demerouti, 2017; Yin et al., 2018). This may be the case for numerous reasons, such as the level of control individuals have in their role, the prestige of their role, the extent to which the demand benefits them, etc. (Bakker and Demerouti, 2017). This being the case, we remain open to the possibility that demands and/or resources may have apparently counter-intuitive effects.

#### JD-R and Learning and Instruction

Although JD-R is centered on workplace processes, it is evident the same factors and processes implicated in workplace functioning are implicated in students’ learning. There are specific contextual factors in academic learning that help or hinder students’ educational outcomes (Martin and Marsh, 2009). This being the case, job demands in the educational setting refer to aspects of learning that require psychological or physical exertion (e.g., performing under a heavy study load and meeting multiple due dates) and are linked with psychological or physical costs (e.g., stress, dropout, and underachievement). Correspondingly, job resources in the educational setting are aspects of learning that help students attain desired academic goals and growth (e.g., teacher/instructional support) and are linked with positive outcomes (e.g., engagement and achievement). In relation to personal resources, in line with Collie et al. (2020a), adaptability can be considered a modifiable capacity that can help students navigate change and effect positive learning outcomes. Indeed, there may also be a buffering role for adaptability in the learning context such that adaptable students may be less likely to experience the negative effects of job demands.

Thus, although JD-R theory is a well-established approach for understanding employees’ workplace functioning (Bakker and Demerouti, 2017), we propose it can also be applied to learning and instruction. Moreover, although there is substantial research harnessing JD-R to investigate teachers’ workplace experiences (e.g., Collie et al., 2020a; Granziera et al., 2021), there is significant scope for investigating the same dynamics among school students.

#### Demands and Resources in the Present Study

In addition to our focus on *adaptability* (as a personal resource), our study comprised one job demand and two job resources. The job demand, *online learning barriers*, refers to the challenges that students experience when learning online at home. It is well documented that factors, such as unreliable internet, difficulties accessing appropriate computing and technology, and distracting home environments, present barriers to students’ online learning (Peña-López, 2015; Australian Academy of Science, 2020). In relation to job resources, *online learning support* refers to the quality of the online learning resources and learning opportunities made available to students by their schools (Yukselturk and Bulut, 2007; Means et al., 2009; Escueta et al., 2017; Gregori et al., 2018; AITSL, 2020). The other job resource is *parent/home help*, which refers to the extent to which parents provide help with schoolwork and the necessary routines and resources are available at home to assist learning (Galpin and Taylor, 2018).

Although we hypothesize that online learning barriers (job demand) will yield negative effects and that online learning support and parent/home help (job resources) will yield positive effects, we are open to the possibility that this may not be so—in keeping with recent developments in JD-R theory stating that there is variability between individuals in how they perceive demands and resources (Bakker and Demerouti, 2017; Yin et al., 2018; Han et al., 2020). Indeed, recent research by Martin et al. (2021) showed that students in high school science perceive and experience a difficult task in different ways, some seeing it as a challenge and some seeing it as a threat. In the case of the present study we might ask, at what point does parent/home help move from being supportive (yielding a positive motivational effect) to being controlling (yielding a negative motivational effect; Neubauer et al., 2020)?

In terms of JD-R’s contended *buffering* effect, we can model the interaction between adaptability and online learning barriers to ascertain the extent to which adaptability may moderate the negative effects of job demands (Collie et al., 2020a; Granziera et al., 2021). These factors are all demonstrated in Figure 1 as key predictors of student outcomes that take the forms of online learning self-efficacy and end of year test achievement—links now discussed.

#### Linking the Resources and Demands With Online Learning Self-Efficacy

Collie et al. (2020a) argued that the nature of individuals’ demands and resources impacts their domain-specific efficacy, which in turn impacts important outcomes, such as performance. *Online learning self-efficacy* refers to students’ perceived and experienced competence in online learning. A large body of research has demonstrated the importance of perceived efficacy for a range of outcomes, including performance (e.g., Bandura, 1997; Martin, 2007, 2009; Klassen and Chiu, 2010; Marsh and Martin, 2011). In JD-R models, the positioning of efficacy can differ, with some models placing it as a personal resource alongside job demands and resources (e.g., Xanthopoulou et al., 2007), while others having efficacy predicted by demands and resources—but notably still referring to it as a personal resource (Collie et al., 2020a). We adopt the latter position because (in line with Collie et al., 2020a) we wanted to focus on what demands and resources lay a foundation for online learning self-efficacy given it is a desirable outcome in itself (as well as being a means to desirable ends, such as achievement; Collie et al., 2020a). Indeed, other researchers have also identified perceived efficacy as an outcome of job demands, job resources, and other personal resources (e.g., Chang, 2013).

Of particular interest in our research is the role of adaptability in predicting online learning self-efficacy. According to Collie et al. (2020a); see also Collie and Martin (2016), adaptability fosters mastery and efficacy experiences—and their research among teachers demonstrated precisely this. Accordingly, we hypothesize that adaptability during times of such uncertainty, variability, and novelty (viz. online learning during COVID-19) will be associated with higher levels of online learning self-efficacy. In addition to this, we suggest that the presence of online learning barriers (job demands) will lead to lower online learning self-efficacy, whereas job resources in the forms of online learning support and parent/home help will be associated with higher online learning self-efficacy.

#### Achievement as an Outcome of Online Learning Self-Efficacy

In most JD-R models, workplace outcomes reflected in diverse forms of performance (e.g., retention and achievement) are the final part of the process (though, the process is cyclical over time; Collie et al., 2020a). Extrapolating to learning and instruction processes under a JD-R framework, academic achievement is contended as an analogous performance outcome (see Figure 1). Thus, the final part of the process examined in our hypothesized model considers the association between online learning self-efficacy and subsequent achievement. This component is also supported by conceptualizing from social cognitive theory (Bandura, 1997) and supported by a long line of empirical research in education (Martin, 2007, 2009; Lee et al., 2014; Schunk and DiBenedetto, 2014). We therefore hypothesize a positive link between online learning self-efficacy and achievement. Moreover, given our focus on adaptability as a predictor of online learning self-efficacy, we also explore the indirect association between adaptability and achievement *via* online learning self-efficacy.

### Mathematics: The Subject Area for This Investigation

For several reasons, mathematics was our focus for this investigation. There is evidence of declining achievement and participation in high school mathematics in Australia (e.g., Thomson et al., 2016; OECD, 2018). There are also concerns that first-year university STEM students are not sufficiently prepared for the level of mathematics skill required at the tertiary level (Nicholas et al., 2015). It is also the case that students can struggle with online formats in mathematics. For example, when assessing online and paper-based tests, Backes and Cowan (2019) found paper-based tests yielded higher mathematics results than online tests. Hassler Hallstedt et al. (2018) found that engaging with a mathematics program on a tablet yielded a small positive effect size for basic arithmetic, but not for arithmetic transfer and problem solving; they also found the positive effects faded over the course of 6 to 12 months. Notwithstanding this, other research has found more positive evidence for online mathematics learning (e.g., Sung et al., 2017). Taken together, mathematics is an area of national priority and one for which there is mixed evidence for effective instruction in online modes. It is, thus, a potentially illuminating focus for research investigating factors that may assist students’ online learning experiences.

### The Role of Salient Background Attributes

In assessing the unique effects of demands and resources, it is important to account for the following background attributes (covariates) that are known to be associated with one or more of this study’s substantive variables: age, gender, language background, parent education, mathematics self-efficacy, and prior mathematics achievement. Older students seem to achieve more highly in technology-assisted learning (Escueta et al., 2017; Sung et al., 2017). Girls tend to score higher in the self-regulatory attributes (Martin, 2007) important for self-directed/autonomous remote online learning (Kirschner and De Bruyckere, 2017). Ethnicity has been found to moderate the effects of online learning on achievement (Nguyen, 2015). In periods of remote learning during COVID-19, parents have struggled with the motivational and learning demands placed on them (Garbe et al., 2020) and unfamiliarity with these processes may be greater for parents with fewer years of education themselves. Online learning self-efficacy and achievement in mathematics are likely to be associated with self-efficacy in mathematics more generally (not just in its online aspects) and also with prior mathematics achievement (e.g., Hattie, 2009).

## Aims of the Present Study

Drawing on JD-R theory and set during a period of COVID-19 entailing fully or partially remote online learning, this research investigated the role of adaptability in high school students’ online learning self-efficacy in mathematics and their end of year mathematics achievement. Following our review of theory and prior research, we pose numerous hypotheses and a research question. Hypothesis 1: beyond the effects of online learning demands, online and parental learning support, and background attributes, adaptability will be positively associated with students’ online learning self-efficacy and gains in end of year achievement. Hypothesis 2: beyond the effects of adaptability, online learning demands, online and parental learning support, and background attributes, online learning self-efficacy will be positively associated with gains in end of year achievement. Hypothesis 3: online learning self-efficacy will significantly mediate the relationship between adaptability and gains in end of year achievement. Research Question 1: what is the role of adaptability in buffering the potentially negative effects of online learning barriers.

## Materials and Methods

### Participants

The sample comprised 1,548 Australian high school students from nine schools. All schools were in the independent school sector and located in or around major urban areas of the state of New South Wales (NSW) on the east coast of Australia. Of the nine schools, four were co-educational, two were single-sex boys’ schools, and three were single-sex girls’ schools. Just over half (53%) of students were boys. Students were in Year 7 (21%), Year 8 (34%), Year 9 (17%), and Year 10 (28%)—the first 4 years of high school in Australia. The average age was 13.77 years (*SD* = 1.16 years). Fourteen percent of students spoke a language other than English at home. Students tended to be from educated backgrounds, with parents/carers scoring 5.19 (*SD* = 1.77) on a scale of 1 (no formal education) to 6 (university education).

### Procedure

The lead researcher’s university provided human ethics approval. School principals then provided approval for their school’s participation. Subsequently, parents/carers and participating students provided consent. An online survey and mathematics test were administered during school hours in the second term (of four school terms) of 2020. As described in the introduction, this was during a period of COVID-19 that entailed fully or partially remote online learning. The end of year online mathematics test was administered in the final term of 2020 when all students had returned to school for in-class lessons. Students were asked to complete the survey and tests on their own.

### Materials

Our substantive factors included job demands, job resources, personal resources, online learning self-efficacy, and performance. Descriptive, reliability, and factor analytic statistics are presented in Table 1. We also assessed background attributes as covariates, comprising age, gender, parent education, and language background.

**Table 1:** Descriptive and measurement statistics.

	Possible range	*M*	*SD*	Reliability (omega)	CFA loading *M*
Online learning barriers (job demands)	0–3	0.217	0.476	–	–
Online learning support (job resources)	1–5	3.711	0.708	0.795	0.659
Parent/home help (job resources)	1–5	2.678	0.856	0.751	0.612
Adaptability (personal resources)	1–7	5.471	1.054	0.800	0.749
Online learning self-efficacy	1–4	2.888	0.910	0.700^†^	0.837
End of year test achievement	0–10	5.745	1.948	–	–

All measures are in relation to mathematics; *M*, mean; *SD*, standard deviation; CFA, confirmatory factor analysis; dash, formative score/single-item indicator.

† reliability estimated for this single item indicator and used to generate error-adjusted score.

#### Job Demands, Resources, and Outcomes

JD-R factors comprised job demands (online learning barriers), job resources (online learning support, parent/home help), personal resources (adaptability), a buffering factor (online demands x adaptability), efficacy (online learning self-efficacy), and performance (end of year achievement test)—all in relation to mathematics. Descriptive and measurement statistics are shown in Table 1. *Online learning barriers* were a formative sum (from 0 to 3) representing the accumulation of barriers to students’ online learning at home, including unreliable Internet, inadequate computing/technology, and little/no access to a quality area for concentration. *Online learning support* comprised five items asking students about the quality of support/resourcing for their online learning (e.g., “How satisfied are you with your online learning platform for mathematics?”), rated on a scale from 1 (very dissatisfied) to 5 (very satisfied). Because the nature of online learning elements (e.g., online learning platforms, such as learning management systems) can be quite variable (Tinmaz and Lee, 2020)—e.g., qualitative responses in the present study revealed more than 20 online learning platforms were used—a given online learning element may not necessarily be a resource per se. Thus, to better ensure we were assessing it as a resource, we asked students to appraise the resource *via* ratings of satisfaction. While we acknowledge resources under JD-R are often assessed in terms of the characteristics or attributes of the resource, we adapted this to assess it in a more nuanced and targeted fashion to establish it more clearly as a resource. In fact, the idea to tap into appraisals of job demands and resources is now being recognized, with researchers suggesting it is only then that the help or hindrance dimension of a job resource/demand can be assessed (Liu and Li, 2018; Ma et al., 2021). *Parent/home help* comprised five items asking about the help they received at home for their learning (e.g., “How often do your parents or someone else in your home help you with your mathematics homework?”), rated on a scale of 1 (never/hardly ever) to 5 (every day/almost every day). *Adaptability* comprised three items (using the Adaptability Scale—Short; Martin et al., 2016) asking students about the extent to which they could adjust their behavior, thinking, and emotion to effectively navigate novelty, variability, and uncertainty (e.g., “In mathematics, to assist me in a new situation, I am able to change the way I do things”), rated on a scale of 1 (strongly disagree) to 7 (strongly agree). *Buffering* was assessed *via* the interaction of online learning demands and adaptability (an interaction term generated through the cross-product of the two zero-centered main effects; Aiken et al., 1991).

*Online learning self-efficacy* was a single item asking students about their perceived competence in online learning (“Overall, how confident are you as an online learner in mathematics?”), rated on a 1 (not confident) to 4 (very confident) scale. Given this was a single-item factor, we sought to account for measurement error by creating an error-adjusted score using the following equation: $\sigma_{h}^{2}$ × (1 − ω_h_), where $\sigma_{h}^{2}$ is the variance of our online learning self-efficacy variable (0.827) and ω_h_ was the reliability of the same variable (Cole and Preacher, 2014; Kline, 2016), which we conservatively estimated at 0.70 in this study. In so doing, unreliability was accounted for in this factor, as would be the case if we had multiple items and estimated a latent factor. This error-adjusted score was used in the confirmatory factor analysis (CFA) and structural equation modeling (SEM; described below). *End of year achievement* was assessed *via* a 10-item mathematics test and operationalized as a formative summed score. Achievement scores were standardized by year level (*M* = 0; *SD* = 1). Questions were structured in 4-answer multiple-choice format, graduated in difficulty and designed to assess underlying mathematical competencies (as opposed to knowledge recall) from the Australian National Curriculum (Kindergarten-10), and associated state syllabus outcomes (e.g., addition, subtraction, patterns, algebra, time, fractions, decimals, percentages, ratio, probability, and area). An example question was “Which of the following is correct? (A: 0.0409 > 0.041, B: 0.21 > 0.200, C: 0.00004 > 0.0003, and D: 0.123 > 0.124),” to assess a part of the syllabus material covering decimals, fractions, and percentages.

#### Background Attributes

In assessing the unique effects of demands and resources, it is important to account for numerous background attributes in modeling. For these background attributes, participants reported age (a continuous measure), gender (0 = male and 1 = female), language background (0 = English speaking and 1 = non-English speaking), and parent education (scale from 1 = no formal education to 6 = university education). Descriptive statistics for these are presented in Participants section, above. We also assessed mathematics self-efficacy (single item from the domain-specific version of the Motivation and Engagement Scale High School Short, Martin, 2020; validated by Martin et al., 2020): “I believe I can do well in mathematics” rated (1 = strongly disagree to 7 = strongly agree; *M* = 5.40, *SD* = 1.94) and prior achievement (10-item mathematics test parallel to the end of year test described above; *M* = 5.52, *SD* = 1.86).

### Data Analysis

Confirmatory factor analysis and SEM were the central analyses, conducted with M*plus* version 8 (Muthén and Muthén, 2017). We used the MLR (maximum likelihood robust to non-normality) estimator that provides parameter estimates with standard errors and a chi-square test statistic that are robust to non-normality (Muthén and Muthén, 2017). To assess model fit, a Comparative Fit Index (CFI) and Tucker Lewis Index (TLI) greater than 0.90, a Root Mean Square Error of Approximation (RMSEA) and Standardized Root Mean Square Residual (SRMR) less than 0.08 indicated acceptable fit (Hu and Bentler, 1999; Kline, 2016). Missing data were dealt with using the M*plus* default, Full Information Maximum Likelihood (FIML; Arbuckle, 1996).

For the CFA, the following factors were included: online learning barriers (formative score), online learning support (latent factor), parent/home help (latent factor), adaptability (latent factor), online learning self-efficacy (error-adjusted score), end of year achievement (formative summed score), and background attributes (each a single indicator, with loading set at 1.00 and residual at 0)—thus, a 12-factor CFA.

The hypothesized structural model (Figure 1) was tested using SEM. In this model, (a) online learning demands, online learning support, parent/home help, adaptability, the interaction of online demands and adaptability (buffering effect), and all background attributes predicted online learning self-efficacy and in turn, (b) these factors—including online learning self-efficacy—predicted end of year achievement (thus, a “fully-forward” model). Because we included prior achievement as a predictor in the model, we could interpret paths to end of year achievement in terms of gains (or declines). Our data also enabled tests of indirect (mediation) effects which were conducted in subsidiary analyses. A parametric bootstrapping approach was used to test mediation. Here, we explored the extent to which online learning self-efficacy mediated the relationship between the various demands and resources and students’ end of year achievement. Analyses were based on bootstrapped standard errors with 1,000 draws (MacKinnon et al., 2002; Shrout and Bolger, 2002).

## Results

### Confirmatory Factor Analysis and Correlations

The 12-factor CFA tested the dimensionality and measurement properties underlying the hypothesized model and also generated bivariate correlations that were the first insight into the relationships of interest in Figure 1. This CFA yielded an acceptable fit to the data, *χ*^2^ (152) = 453.25, *p* < 0.001, CFI = 0.956, TLI = 0.933, RMSEA = 0.036, and SRMR = 0.033. Factor loading means are shown in Table 1 and correlations are presented in Table 2. Here, we summarize only significant correlations among substantive factors that are key to the hypothesized model (all other significant and non-significant correlations are in Table 2). The following were significantly correlated with online learning self-efficacy: online learning barriers (*r* = −0.247, *p* < 0.001), online learning support (*r* = 0.689, *p* < 0.001), parent/home help (*r* = 0.153, *p* < 0.001), and adaptability (*r* = 0.529, *p* < 0.001). Thus, online learning barriers were associated with lower online learning self-efficacy, whereas online learning support, parent/home help, and adaptability were associated with higher online learning self-efficacy. The following were significantly correlated with end of year achievement: online learning self-efficacy (*r* = 0.256, *p* < 0.001), online learning barriers (*r* = −0.097, *p* < 0.001), online learning support (*r* = 0.140, *p* < 0.001), parent/home help (*r* = −0.090, *p* < 0.01), and adaptability (*r* = 0.272, *p* < 0.001). Thus, online learning barriers and parent/home help were associated with lower end of year achievement, whereas online learning self-efficacy, online learning support, and adaptability were associated with higher end of year achievement.

**Table 2:** Correlations from CFA.

		1	2	3	4	5	6	7	8	9	10	11	12
JD-R factors													
1.	Online learning barriers	–	−0.239^***^	−0.058	−0.152^***^	−0.247^***^	−0.097^***^	0.038	0.008	−0.044	0.024	−0.149^***^	−0.114^***^
2.	Online learning support		–	0.080^*^	0.408^***^	0.689^***^	0.140^***^	0.050	−0.054	0.031	0.018	0.258^***^	0.167^***^
3.	Parent/home help			–	0.230^***^	0.153^***^	−0.090^**^	−0.177^***^	−0.077^**^	0.082^**^	0.040	0.166^***^	−0.096^**^
4.	Adaptability				–	0.529^***^	0.272^***^	−0.111^***^	−0.200^***^	0.063^*^	0.047	0.556^***^	0.263^***^
5.	Online learning self-efficacy					–	0.256^***^	−0.107^**^	−0.098^**^	0.108^**^	0.047	0.406^***^	0.235^***^
6.	End of year achievement						–	−0.029	−0.097^***^	0.174^***^	0.144^***^	0.308^***^	0.561^***^
Background attributes													
7.	Age							–	0.074^**^	−0.019	0.039	−0.066^**^	−0.012
8.	Gender (M/FM)								–	−0.021	−0.060^**^	−0.193^***^	−0.147^***^
9.	Parent education									–	0.030	0.116^***^	0.161^***^
10.	NESB										–	0.061^**^	0.153^***^
11.	Math self-efficacy											–	0.309^***^
12.	Prior achievement												–

All JD-R factors are in relation to mathematics; NESB, non-English speaking background; M, male; and FM, female.

* *p* < 0.05,

** *p* < 0.01, and

*** *p* < 0.001.

### Structural Equation Modeling

**Figure 2:**
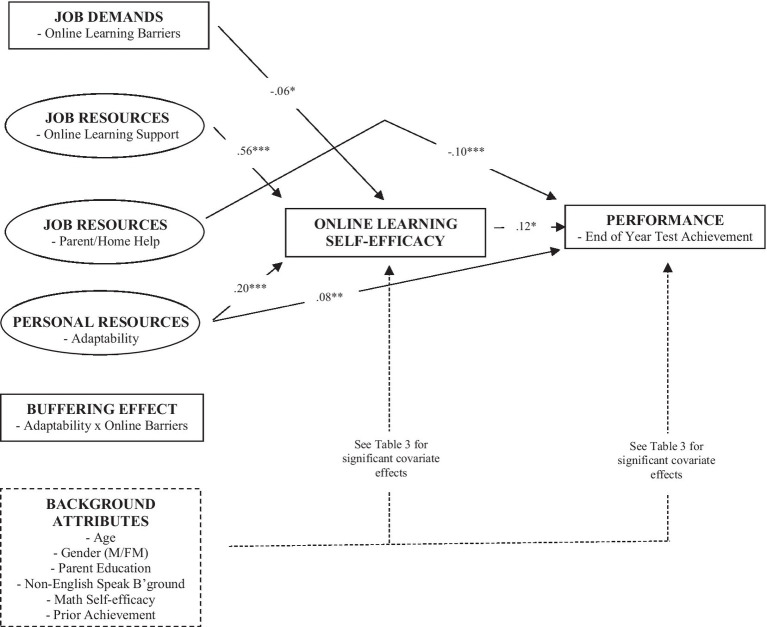
Standardized beta coefficients for JD-R process in online mathematics. All JD-R factors are in relation to mathematics; ^*^p < 0.05, ^**^p < 0.01, and ^***^p < 0.001. See Table 3 for indirect and covariate effects.

We then tested the model in Figure 1 using SEM. This yielded an acceptable fit to the data, *χ*^2^ (163) = 509.76, *p* < 0.001, CFI = 0.949, TLI = 0.921, RMSEA = 0.037, and SRMR = 0.036.^1^ Table 3 and Figure 2 show results. Here, we summarize only significant paths among substantive factors. All other significant and non-significant paths are in Table 3. Significant predictors of online learning self-efficacy (beyond the effects of all background attributes) were as follows: online learning demands (*β* = −0.062, *p* < 0.05), online learning support (*β* = 0.562, *p* < 0.001), and adaptability (*β* = 0.202, *p* < 0.001). Thus, online learning demands were predictive of lower online learning self-efficacy, whereas online learning support and adaptability were predictive of higher online learning self-efficacy. In turn, beyond the effects of background attributes, significant predictors of end of year achievement gains were as follows: online learning self-efficacy (*β* = 0.118, *p* < 0.05), parent/home help (*β* = −0.103, *p* < 0.001), and adaptability (*β* = 0.079, *p* < 0.05). Thus, online learning self-efficacy and adaptability were predictive of gains in end of year achievement, whereas parent/home help was predictive of declines in end of year achievement (discussed in further detail below).

**Table 3:** Standardized direct and indirect effects for JD-R process in online mathematics.

	Online learning self-efficacy	End of year test achievement
	*β*	*β*
JD-R factors		
Online learning barriers (job demands)	−0.062^*^	−0.012
Online learning support (job resources)	0.562^***^	−0.072
Home/parent help (job resources)	0.022	−0.103^***^
Adaptability (personal resources)	0.202^***^	0.079^*^
Adaptability × Barriers (buffering)	−0.008	−0.001
Online learning self-efficacy	–	0.118^*^
Background attributes		
Age	−0.101	−0.011
Gender (M/FM)	0.011	0.011
Parent education	0.053	0.079^***^
Non-English speaking background	0.018	0.063^**^
Math self-efficacy	0.117^**^	0.095^**^
Prior achievement	0.039	0.463^***^
Indirect effects		
Online learning barriers → Online learning self-efficacy → End of year test achievement		−0.007
Online learning support → Online learning self-efficacy → End of year test achievement		0.066^*^
Home/parent support → Online learning self-efficacy → End of year test achievement		0.003
Adaptability → Online learning self-efficacy → End of year test achievement		0.024^*^
Adaptability × Barriers → Online learning self-efficacy → End of year test achievement		−0.001
Total effects		
Online learning barriers → Online learning self-efficacy → End of year test achievement		−0.020
Online learning support → Online learning self-efficacy → End of year test achievement		−0.006
Home/parent support → Online learning self-efficacy → End of year test achievement		−0.100^***^
Adaptability → Online learning self-efficacy → End of year test achievement		0.103^***^
Adaptability × Barriers → Online learning self-efficacy → End of year test achievement		−0.002

All JD-R factors are in relation to mathematics.

* *p* < 0.05,

** *p* < 0.01, and

*** *p* < 0.001.

Finally, we examined the indirect paths from demands and resources to end of year achievement gains *via* online learning self-efficacy. There were two significant indirect paths: online learning support → online learning self-efficacy → end of year achievement, *β* = 0.066, *p* < 0.05; adaptability → online learning self-efficacy → end of year achievement, *β* = 0.024, *p* < 0.05. Thus, online learning self-efficacy mediated the relationship between online learning support and end of year achievement gains; it also mediated the relationship between adaptability and end of year achievement gains. Table 3 also presents total effects, showing that adaptability has the largest net positive effect on achievement gains of all predictors (*β* = 0.103, *p* < 0.001), while parent/home help has the largest net negative effect, being significantly associated with achievement declines (*β* = −0.100, *p* < 0.001).

## Discussion

Adaptability is a personal resource that has potential to assist students through times of novelty, variability, and uncertainty—such as what they have experienced during COVID-19. Drawing on JD-R theory and a large sample of Australian high school students, we examined the role of adaptability (a personal resource) in predicting students’ online learning self-efficacy and the role of their online learning self-efficacy in predicting their end of year achievement during a period of COVID-19 that entailed fully or partially remote online learning. We found that adaptability was significantly associated with greater online learning self-efficacy and with gains in achievement (supporting Hypothesis 1); online learning self-efficacy was also significantly associated with gains in achievement (supporting Hypothesis 2)—and significantly mediated the relationship between adaptability and achievement (supporting Hypothesis 3). These effects were significant beyond any variance attributable to online learning demands, online learning support, parent/home help, and background attributes. Our findings therefore confirm the hypothesized role of adaptability as an important personal resource and have practical implications for better supporting students in their online learning, including through periods of remote online instruction, such as during COVID-19.

### Findings of Particular Note

In line with hypotheses, findings showed that adaptability (a personal resource) was significantly associated with greater online learning self-efficacy—beyond the effects of online learning barriers (job demands), online learning support and parent/home help (job resources), and background attributes. In fact, adaptability not only predicted online learning self-efficacy as hypothesized, but also directly predicted gains in end of year test achievement—and significantly indirectly predicted end of year achievement *via* the mediating role of online learning self-efficacy. Adaptability thus presents as an important factor in how students navigate their online learning during periods of significant novelty, variability, and uncertainty (in this case, during a period of COVID-19 that entailed fully or partially remote online learning). We can infer that the adjustments required by students to navigate these uncertain circumstances were well met by the psychological attribute of adaptability. This expands on the pre-COVID-19 evidence base for the positive effects of adaptability on students’ educational outcomes (Martin et al., 2013; Holliman et al., 2018, 2019, 2021). Thus, in line with Collie et al. (2020a); see also Collie and Martin, 2016), it seems that adaptability fosters mastery and efficacy experiences—manifested in our research by online learning self-efficacy.

We can also now add to what we know about factors that may enhance the effectiveness of online learning. As described earlier, there is a mixed evidence base for the effectiveness of online learning modes, representing a diversity of positive effects (Yuwono and Sujono, 2018), negative effects (Peña-López, 2015; Delgado et al., 2018; Clinton, 2019), and null effects (Cavanaugh et al., 2004; Kraft and Hill, 2020). It has been suggested that part of this diversity is due to the variety of factors that influence online learning effectiveness. Research has previously identified factors, such as technology access, technology skills, instructional and resource quality, parent/home support, ethnicity, socioeconomic status, and learning support needs (AITSL, 2020; Australian Academy of Science, 2020). To this, we can now add adaptability which predicted online learning self-efficacy and also achievement *via* online learning self-efficacy. Indeed, because adaptability is a modifiable psychological attribute (Martin et al., 2013; Granziera et al., 2021), it represents a viable direction for assisting students’ online learning experience.

In addition to the positive role of adaptability, online learning self-efficacy was associated with gains in end of year test achievement. Thus, the extent to which students perceived and experienced competence in online learning was important for their subsequent academic performance (beyond prior academic performance). This is consistent with contentions under classic conceptualizing (e.g., social cognitive theory; Bandura, 1997) and research (e.g., Martin, 2007, 2009; Lee et al., 2014; Schunk and DiBenedetto, 2014). Particularly noteworthy is the fact that online learning self-efficacy predicted gains in achievement beyond the effects of general mathematics self-efficacy on achievement—thus, students’ efficacy in online mathematics learning itself (net general mathematics self-efficacy) was linked to their later mathematics achievement. These findings demonstrate that achievement is not only a function of subject-specific mathematics self-efficacy (consistent with prior research; Green et al., 2007) but also a function of domain-specific efficacy within the subject: in this case, online learning self-efficacy.

### Unexpected Findings of Note

Following prior research among teachers, we modeled the interaction between personal resources (adaptability) and job demands (online learning barriers) to ascertain the extent to which adaptability may buffer the negative effects of job demands (Collie et al., 2020a; Granziera et al., 2021)—to address Research Question 1. This interaction (buffering) effect was not statistically significant; instead, it was the main effects of adaptability (positive effect) and online learning barriers (negative effect) that predicted online learning self-efficacy. This is nonetheless important, as it shows that adaptability yields a positive effect beyond the barriers that students experience in online learning. Thus, adaptability surmounts the negative effects of online learning barriers, even if it does not buffer them.

It was also initially surprising to identify a negative path between parent/home help and end of year test achievement—higher levels of help from parents at home were associated with lower end of year achievement. We suspect this may be explained by the reality that academically struggling students are likely to require more help from their parents—thus, lower achieving students reported higher levels of parent/home help. But how do we reconcile this with other research showing that low parental involvement is associated with lower achievement (e.g., Lara and Saracostti, 2019)? We cannot rule out the possibility that the more intense parental involvement with their adolescent child while at home during COVID-19 may have been perceived by the student as controlling and giving rise to a reduction in autonomy-supportive parenting practices (e.g., Neubauer et al., 2020)—leading to reduced achievement. Further research is needed to understand this better, but it does align with recent developments in JD-R theory and research identifying variability between individuals in how they perceive demands and resources (Bakker and Demerouti, 2017; Yin et al., 2018; Han et al., 2020; Martin et al., 2021), with some seeing resources as more a hindrance than a help. In the case of our study, perhaps there was a controlling role for parent/home help that was perceived as a hindrance, and which evinced a negative effect for achievement. Similar apparently counter-intuitive effects of parental involvement and attitudes on students’ academic outcomes have been found in other studies. Murayama et al. (2016) suggested that overly positive parental judgments may be disadvantageous because they are associated with over involvement, controlling behavior, and excessive pressure. Other studies explore parental “intrusive support” of students. For instance, Gunderson et al. (2012) explain how expectations of parents, based on their own anxieties and stereotypical beliefs, can lead to lower achievement, *via* intrusive support during homework. Furthermore, we suggest it is important to better understand the nature and impact of parental involvement as relevant to the COVID-19 pandemic itself. For example, additional research is needed to explore diverse dimensions of parental involvement in their children’s schoolwork during the pandemic with particular interest in the factors that determine whether this involvement is perceived as a help or a hindrance.

There were two non-significant main effects also worth noting (but they were not the substantive focus and we did not formulate hypotheses for them): a non-significant predictive path between online learning barriers and achievement and a non-significant predictive path between online learning support and achievement. We suggest this is noteworthy because these two predictors were significantly correlated with achievement (see Table 2), but after including the significant predictive roles of adaptability and parent/home help on achievement, online learning barriers and support explained no further variance in achievement. Moreover, because adaptability yielded a unique net positive effect on achievement relative to the net negative effect of parent/home help (see total effects in Table 3) and because adaptability shared more variance with online learning barriers and support than did parent/home help (see Table 2), we suggest it is the presence of adaptability that played a major role in mitigating the predictive paths from online learning barriers and support to achievement. The two non-significant paths also underscore an important mediating role for self-efficacy, in similar vein to prior research finding that teacher self-efficacy fully mediates the link between teachers’ adaptability and students’ outcomes (Collie et al., 2020a). These findings, we suggest, further highlight the importance of considering adaptability as a personal resource in JD-R models generally (in line with emerging research: Collie et al., 2020a; Granziera et al., 2021), and in models exploring disruptive circumstances, such as COVID-19 more specifically.

### Implications for Theory and Practice

Based on the findings, we believe we have successfully adapted JD-R theory to the (online) learning and instruction setting in high school mathematics. We showed that personal resources by way of adaptability positively impacted students’ online learning experiences and outcomes (consistent with research showing the positive impacts of adaptability among teachers; Collie et al., 2020a; Granziera et al., 2021). We showed that job demands by way of online learning barriers were associated with lower online learning self-efficacy (consistent with research showing such barriers impede online learning; e.g., Peña-López, 2015; Australian Academy of Science, 2020). We also showed that job resources by way of online learning support and parent/home help were associated with higher online learning self-efficacy (consistent with prior research demonstrating a supportive role for these factors; e.g., Yukselturk and Bulut, 2007; Means et al., 2009; Escueta et al., 2017; Galpin and Taylor, 2018; Gregori et al., 2018; AITSL, 2020).

The salient role of adaptability in this study also suggests it as an important point for educational intervention. As adaptability is an emerging area of research, suggested practice directions have drawn on existing related frameworks, such as the resilience research by Rutter (1987) and Morales (2000). For example, Martin et al. (2013); see also Burns and Martin (2014) and Martin and Burns (2014) identified the following steps to boost students’ adaptability: (1) teach students how to recognize novelty, variability, and uncertainty, (2) explain to students how they can adjust their behavior, thinking, and/or emotion to navigate the novelty, variability, and uncertainty (strategies are detailed below), (3) encourage students to recognize the benefits of these psycho-behavioral adjustments, and (4) explain to students that continued behavioral, cognitive, and/or emotional responses to novelty, variability, and uncertainty represent the “adaptability cycle” and that this cycle leads to enhanced ongoing positive outcomes in the face of change.

Burns and Martin (2014) and Martin and Burns (2014) propose that the second step of this process (adjusting behavior, cognition, and emotion) is the most critical part of the adaptability cycle. According to Martin (2014); see also Burns and Martin (2014) and Martin and Burns (2014) and extrapolating his guidance to online learning, (a) students can adjust their cognition by thinking about a new online task in a different way (e.g., considering the opportunities the new online option might offer); (b) students can adjust their behavior by seeking out new or more online information and resources, or asking for help (e.g., asking a teacher to help with a new online learning management system); and (c) students can adjust their emotions by minimizing negative feelings (e.g., frustration) when they need to juggle in-class and online learning modes (e.g., choosing not to focus on disappointment if the teacher engages an online learning approach that is not to the student’s preference).

Our findings also showed that adaptability is not the only practical implication to take from this study; it is also important to remove barriers to students’ online learning and to enhance their online learning resources. Attending to the online learning barriers would entail addressing Internet and connection issues, ensuring students have access to appropriate computing and technology, and identifying places for them to engage with online learning so they can concentrate (Australian Academy of Science, 2020). Attending to online learning support would involve ensuring high quality learning management systems, providing ample opportunity to interact with and receive help from the teacher online, and being provided with the opportunity to engage with peers online but also to work independently as appropriate.

### Limitations and Future Directions

There are some limitations in this study that are important to take into account when interpreting the findings and which also have implications for future research into online learning. First, our correlational research data cannot be interpreted as supporting causal conclusions. Experimental work that manipulates adaptability and explores for any subsequent shifts in online learning self-efficacy would better establish (or not) the causal role of adaptability. Indeed, Galpin and Taylor (2018) and others (e.g., Means et al., 2009; Patrick and Powell, 2009; Quesada-Pallarès et al., 2019) recommend more studies that can test causality (including experimental studies) and the factors that may moderate whether online learning is beneficial or not. Second, although our achievement data were based on a mathematics test tapping into diverse aspects of mathematics syllabus, it will be important to expand the outcome measures to assess other aspects of mathematics performance. Third, there tends to be more research into online learning among post-school students (e.g., university/college) and to some extent among high school students (such as in our study); there is a need for more research among elementary school students (Means et al., 2009; Clinton, 2019). Fourth, this study relied on student reports of online learning barriers and support. Additional indicators, such as parent and teacher ratings, might be used in future to triangulate findings with students’ reports of constructs in our study. Also on the matter of measurement, we assessed online learning resources in terms of student appraisals (*via* ratings of satisfaction) and not in terms of characteristics of the resources themselves. Findings and conclusions regarding job resources in our study must take this into account. Fifth, we suggest research that can identify different combinations of demands and resources and their relationships to online learning self-efficacy and academic achievement. As a case in point, latent profile analysis may identify distinct typologies of students who balance the diverse online demands and resources in different ways. Prior JD-R research has conducted latent profile analysis among teachers (Collie et al., 2020b) and expanding this to students would be illuminating.

Sixth, it will be helpful to understand adaptability and its role in online learning in real-time. For example, research has identified the in-situ dimensions of students’ learning and engagement (Schneider et al., 2016; Martin et al., 2020); online learning demands and resources are also likely to have salient in-situ aspects. Seventh, due to constraints of time and to accommodate the fact students were located in diverse combinations of online and in-class learning modes, we wanted to guard against asking extensive batteries of questions about their online experience. Thus, single-item indicators were used in some cases. Although there is research suggesting single-item scales have merit in cases where long scales are not able to be used (e.g., Gogol et al., 2014) and we modeled an error-adjusted score for our central online learning self-efficacy factor, future research might look to administering more extensive item sets. Eighth, our research was set in mathematics which is a challenging school subject and one in which students can struggle (Thomson et al., 2016; OECD, 2018). To the extent this is so, there may be disproportionate challenges in online mathematics learning—or, it may emerge there are unique opportunities afforded to mathematics when in online learning modes. It is thus important to expand the present study to other school subjects. Ninth, students in our sample were from above average SES backgrounds. As such, these students likely had fewer online learning barriers and more online learning support than some other cohorts of students. Our findings may be just the tip of the iceberg in terms of the role of these demands and resources. Finally, online learning platforms, programs, and content tend to be developed and published faster than research can assess their effectiveness (Escueta et al., 2017)—signaling a need to conduct more rapid research in order for researchers and research to stay abreast of the fast pace of developments in online learning.

## Conclusion

The COVID-19 pandemic necessitated a rapid shift to remote learning for students around the world. During this time, in-class learning and instruction moved to remote online modes at speed and scale. Harnessing JD-R theory, the present study identified adaptability as a personal resource that may support students’ online learning experience and achievement during such times. Findings demonstrated that adaptability does indeed play a significant role in this process, and thus may be an important personal resource to foster in students’ online learning during COVID-19—and beyond.

## Data Availability Statement

The datasets presented in this article are not readily available because consent from participants to share dataset is not available; summative data (e.g., correlation matrix with standard deviations) are available to enable analyses. Requests to access the datasets should be directed to Andrew Martin, andrew.martin@unsw.edu.au.

## Ethics Statement

The studies involving human participants were reviewed and approved by the UNSW Human Ethics Committee. Written informed consent to participate in this study was provided by the participants’ legal guardian/next of kin.

## Author Contributions

AM shared in the development of the research design and led data analysis and report writing. RC and RN shared in the development of the research design and assisted with data analysis and report writing. All authors contributed to the article and approved the submitted version.

## Conflict of Interest

The authors declare that the research was conducted in the absence of any commercial or financial relationships that could be construed as a potential conflict of interest.

## Publisher’s Note

All claims expressed in this article are solely those of the authors and do not necessarily represent those of their affiliated organizations, or those of the publisher, the editors and the reviewers. Any product that may be evaluated in this article, or claim that may be made by its manufacturer, is not guaranteed or endorsed by the publisher.

## Footnotes

^1^Because there were diverse modes of online mathematics instruction during this period of COVID-19 (e.g., different combinations of class-based/online learning, small group learning, and solo learning on any given school day), students were not always nested in their mathematics classrooms. Thus, we did not statistically account for clustering/nesting in our analyses. For completeness, however, when we tested the hypothesized model (Figure 1) using the M*plus* Type = Complex command (adjusting standard errors for the nesting of students within classrooms), we derived good fit (*χ*^2^ [163] = 493.38, *p* < 0.001, CFI = 0.948, TLI = 0.919, RMSEA = 0.036, and SRMR = 0.036) and the same substantive effects as our unadjusted model, with the minor exception that the online learning barriers → online learning self-efficacy path was significant at *p* < 0.10 (*β* = −0.062, *p* = 0.067), not *p* < 0.05.
